# Current situation and distribution equality of public health resource in China

**DOI:** 10.1186/s13690-020-00474-3

**Published:** 2020-09-22

**Authors:** Honghui Yao, Chaohong Zhan, Xinping Sha

**Affiliations:** 1 Department of Learning, Informatics, Management and Ethics, Karolinska Institute, 171 77 Stockholm, Sweden; 2 Department of Neurosurgery, Xiangya Hospital, Central South University, Changsha, 410008 Hunan Province China; 3 Department of infectious disease, Xiangya Hospital, Central South University, Changsha, 410008 Hunan Province China; 4 Xinagya Changde Hospital, Changde, 415000 Hunan Province China

**Keywords:** Public health resource, Inequality, Theil

## Abstract

**Background:**

The public health system has been developed in China for several years while no research explores its distribution. This research aims to describe the variation and equality of public health allocation from 2013 to 2018 and explore the source of inequality.

**Methods:**

Data in this research was obtained from the China Health Statistics Yearbook 2014 to 2019 and the China Statistical Yearbook 2019. Four indicators were chosen in describing the development and current situation of the public health system. Three of them were used to evaluate allocation equality. 31 provinces were categorized into western, middle, and eastern groups based on geographical and economic conditions. Total allocation equality, inter- and intra-difference were all measured by the Theil index.

**Results:**

All indicators showed a stably upwards trend except for the number of public health institutions. The allocation gap of the public health institution per km^2^ was larger than that per 10,000 capita. Theil index of three indicators continually rose from 2013 to 2018 and the inequality of public health institutions allocation was the highest one. The western region had the highest Theil index in technical personnel and beds allocation. Among the three regions, the western region contributed most to inequality.

**Conclusions:**

The public health workforces and institutions are still under the requirement of the National Medical and Health Service System Plan. From 2013 to 2018, the equality of public health resources stably decreases, which is mainly contributed by the internal difference within the western region. Further research should be done to explore the possible cause of the results. Problems founded in this research should be solved by multisectoral cooperation.

## Background

Public health system is usually defined as “all public, private, and voluntary entities that contribute to the delivery of essential public health services within a jurisdiction” [1]. In the past 71 years, China has got enviable success in building a completed public health system. The earliest Chinese public health practice can be dated back to the 1920s when the researchers investigated the shortage of drug supply in rural areas [2]. After 1949, the Chinese government confronted with severe situations in public health – lacked health resources and high epidemics of infectious diseases [3]. To solve these problems, village doctor training (also known as barefoot doctors) and disease prevention were launched, aimed to provide public health services and basic medical services as well as basic vaccinations [4]. The population health in China improved a lot from 1952 to 1982 because of these policies, for instance, the average life expectancy sharply increased from 35 to 68 years old and the infant mortality rate decreased from 250 to 40 deaths per 1000 live births [5].

However, the transformation from the planning economy to the market economy challenged the Chinese public health system between the 1980s and 1990s [4]. The insufficient government funding pushed the public health institutes to guarantee their revenues by providing services, which reduced their disease prevention and control functions [6]. Besides, nearly 1/3 public health institutes in rural areas were devastated and 1/3 struggled to survive during this period [7]. The brain drain, outdated equipment, unqualified staff were also straits for public health institutions [8]. Though the Chinese government established the Bureau of Endemic Prevention and Control and the original Chinese Center for Disease Control and Prevention in 1986 and 2002, the situation of public health did not improve [2, 9].

The broken out of severe acute respiratory syndrome (SARS) revealed the weakness in the Chinese public health system, thus the government made numerous corrections to amend it [10]. For instance, community health centers (CHCs) were established in 2005 to provide primary care, rehabilitative services, and prevention [11]. The execution of the new health system reform in China also made a further improvement to the public health system. As one of five priority areas in the 2009 health reform, the Equalization of Basic Public Health Services (EBPHS) was published to promote basic public health services and public health programs through both financial support and delivery [12]. This reform made great achievements. After 2009, the species of public health services expanded to 14 categories and the number of public health programs also increased [4, 12]. Further policies were continually published after Chinese health reform came into “deep zone” in 2013 such as increasing investment and adjusting resource allocation in public health [13]. Besides, the Notice of the General Office of the State Council on Issuing the Outline for the Planning of the National Medical and Health Service System (2015–2020) made a further requirement about public health resource allocation [14].

Nevertheless, the effectiveness of policies is still unknown. Though some studies focused on health resource allocation, they mainly concentrated on medical resource allocation [15, 16]. The public health resources allocation condition still uninvestigated, which impedes the improvement of previous policies. Besides, some flaws about the Chinese public health system appears during this COVID-19 pandemic, such as the shortage of emergency professionals in the public health system and a dearth of funding [17]. The assessment of public health resource allocation equality is able to provide some evidence to remedy those deficiencies.

Health equality assessment is usually employed to evaluate the development of health system financial [18] or healthcare resources allocation [15, 16]. The most common instrument used in health equality evaluation is Lorenz curves and Gini coefficients, which are normally used in measuring horizontal equality [19]. Theil index, Atkinson index, the index of dissimilarity, and measures of association also be employed in evaluation [20]. Up to now, there lacks research in exploring Chinese public health resources development and allocation conditions. Besides, Gini coefficient is incapable of allowing for within or between income group comparisons [19], Atkinson index, and the index of dissimilarity are not suitable in this study because of data limitation. Thus, we described the development of Chinese public health resources from 2013 to 2018 and assess its allocation equality based on the data from the Chinese Health Statistics Yearbooks.

## Methods

### Data resources

In this study, data were obtained from the China Health Statistics Yearbook 2014 to 2019 and the China Statistical Yearbook 2019. 31 provinces, autonomies, and municipalities as well as Xinjiang Uyghur Autonomous Region in mainland China, were included in data analysis. Because of the inconsistency data collection and statistical standard, Taiwan province, Hongkong, and Macao Special Administrative Regions were not involved. These provinces were divided into three groups based on geographical position and the Gross Domestic Product (GDP) per capita [21]. The western region group included Inner Mongolia, Guangxi, Chongqing, Sichuan, Yunnan, Tibet, Gansu, Shanxi, Guizhou, Ningxia, Qinghai, and Xinjiang Uyghur Autonomous Region. Jilin, Anhui, Heilongjiang, Henan, Hubei, Hunan, Jiangxi, as well as Shanxi, were classified as the middle region group. The rest provinces – Tianjin, Beijing, Guangdong, Liaoning, Hebei, Shanghai, Jiangsu, Zhejiang, Shandong, Hainan, and Fujian belonged to the eastern region group.

### Indicators

To describe the trend of public health resources changing from 2013 to 2018, we selected four indicators – the number of public health institutions, the amount of public health technical personnel, the number of beds, and equipment in public health institutions. Due to the availability of data, we only used the number of public health institutions, the amount of public health technical personal, and beds quantity in public health institutions. Detail definitions of four indicators were demonstrated as following:

1. Public health institutions were defined as all included centers for disease control and prevention, maternal and children health care hospitals, specialist disease prevention and treatment hospitals, health education centers, emergency aid centers, blood centers, and health inspection institutions [22].
2. Public health technical personnel constituted by practicing physicians, practicing physician assistants, practicing nurses, pharmacists, technicians, trainee physicians, and other health professionals. The management of public health workers and support technicians were not included in this study [22].
3. Beds in public health institutions referred to the actual amount of beds in these public health institutions we mentioned above, which were composed of formal beds, care beds, simple equipped beds, sterilized beds, repaired beds, and those beds were stopped using because of hospital expansion. The neonatal beds, pre-delivery beds, observation beds, temporary beds, and beds for patients’ relatives were not incorporated in the amounts of beds [22].
4. Equipment in public health institutions referred to the actual number of equipment in public health institutions, including both installed equipment and uninstalled equipment. Only equipment whose price was larger than 10,000 yuan incorporated in this research. However, scrapped device and ordered equipment which did not arrive in institutions were not counted into equipment amounts [22].

The description of the public health resources current situation was based on both population and geographical distribution perspective. The allocation equality of these resources was calculated on the view of population distribution.

### Data analysis

The allocation inequality of public health resources was computed by the Theil index with Stata 16.0. Theil index is an entropy-based measurement that has been used to investigate income inequality originally since it was established in 1975 [23]. Besides income inequality, the Theil index was also developed to explore healthcare allocation inequality [15] and health inequality [24]. The scope of this index is from 0 to 1, the larger Theil index is, the less equitable among different areas will be. The formula of the Theil index is followed: ${T}_T={T}_{\omega }+{T}_B=\left[{\sum}_{j=1}^J{P}_j{T}_j\right]+\left[{\sum}_{j=1}^J{P}_j\ln \left(\frac{P_j}{y_j}\right)\right]$. In this formula, *T*_*T*_ is the total Theil index, which can be divided into *T*_*B*_ and *T*_*ω*_, refer to the total between-group Theil index and total within-group Theil index, respectively. *P*_*j*_ means the population proportion counts for total regional population and *y*_*j*_ represents public health resources accounts for total regional public health resources. Both “within-group” and “between-group” contribution can be computed by dividing T.

## Results

### The development of Chinese public health resource from 2013 to 2018

The change in public health resources from 2013 to 2018 demonstrates in Table 1. The amount of public health technical personal, the number of beds, and equipment increased from 2013 to 2018. With the growth of population, the per capita public health resources also rose except for the quantity of public health institutions. Among these indicators, the number of equipment showed the largest increasing trend. From 2013 to 2018, the number of technical, beds, and equipment per 10,000 capital climbed 8.72, 24.68, and 50.28%, however, the number of public health institutions per 10,000 capital reduceed 43.48%.

**Table 1:** The basic information of total public health resources from 2013 to 2018

Year	Population (10,000 persons)	Public health institutions		Public health technical personnel		Beds in public health institutions		Equipment in public health institutions	
		Number	Per 10,000 persons	Number	Per 10,000 persons	Number	Per 10,000 persons	Number	Per 10,000 persons
2013	136,072	31,155	0.23	608,560	4.47	214,870	1.58	481,148	3.54
2014	136,782	35,029	0.26	631,558	4.62	223,033	1.63	530,587	3.88
2015	137,462	31,927	0.23	639,189	4.65	236,342	1.72	572,371	4.16
2016	138,271	24,866	0.18	646,425	4.68	247,228	1.79	618,857	4.48
2017	139,008	19,896	0.14	661,616	4.76	262,570	1.89	686,572	4.94
2018	139,538	18,033	0.13	678,258	4.86	274,394	1.97	742,759	5.32

### The current situation of Chinese public health resource from 2018

To deeply understand the public health resources allocation, we analyzed it from the perspective of both geographic (per square kilometer) and population (per 10,000 capita) distribution, and the detailed information is put in Table 2.

**Table 2:** Regional distribution of public health resources in 2018

Region	Province	Public health institutions		Public health technical personnel		Beds in public health institutions	
		Per square kilometers	Per 10,000 people	Per square kilometers	Per 10,000 people	Per square kilometers	Per 10,000 people
Western region	Inner Mongolia	0.000	0.193	0.013	6.058	0.004	1.712
	Guangxi	0.005	0.255	0.153	7.333	0.061	2.939
	Chongqing	0.002	0.049	0.133	3.534	0.055	1.464
	Sichuan	0.001	0.084	0.074	4.297	0.027	1.539
	Yunnan	0.001	0.108	0.062	4.894	0.021	1.655
	Tibet	0.000	0.422	0.001	4.285	0.000	1.087
	Gansu	0.003	0.529	0.031	5.273	0.011	1.905
	Shanxi	0.003	0.159	0.115	6.144	0.042	2.239
	Guizhou	0.002	0.095	0.100	4.902	0.048	2.342
	Ningxia	0.001	0.128	0.063	6.077	0.019	1.820
	Qinghai	0.000	0.295	0.004	4.839	0.001	0.839
	Xinjiang	0.000	0.280	0.007	4.799	0.002	1.195
Middle region	Jilin	0.002	0.150	0.062	4.269	0.015	1.069
	Anhui	0.004	0.096	0.123	2.709	0.050	1.114
	Heilongjiang	0.002	0.195	0.038	4.609	0.018	2.111
	Henan	0.010	0.165	0.302	5.252	0.154	2.678
	Hubei	0.003	0.085	0.179	5.637	0.092	2.892
	Hunan	0.004	0.123	0.179	5.499	0.090	2.764
	Jiangxi	0.004	0.159	0.150	5.381	0.084	3.017
	Shanxi	0.003	0.121	0.120	5.032	0.033	1.379
Eastern region	Tianjin	0.008	0.062	0.385	2.785	0.063	0.454
	Beijing	0.007	0.051	0.715	5.577	0.146	1.140
	Guangdong	0.006	0.093	0.363	5.759	0.164	2.597
	Liaoning	0.005	0.154	0.093	3.104	0.025	0.824
	Hebei	0.004	0.091	0.159	3.956	0.072	1.789
	Shanghai	0.017	0.045	1.403	3.646	0.212	0.552
	Jiangsu	0.008	0.100	0.251	3.200	0.073	0.925
	Zhejiang	0.004	0.069	0.257	4.572	0.092	1.628
	Shandong	0.007	0.108	0.328	5.017	0.168	2.575
	Hainan	0.004	0.127	0.173	6.301	0.051	1.870
	Fujian	0.004	0.114	0.139	4.267	0.071	2.172

There were not huge gaps among different provinces for the population distribution perspective. The extent of public health institutions allocation per 10,000 capita was between 0.045 (Shanghai) and 0.529 (Gansu). Hainan province had the largest number of technical personal (6.301), which was 2.3 times than that in Anhui (2.709). Besides, the three indicators do not show great differences among the three regions.

However, the analysis results of resource allocation per square kilometers were opposite to that in the population distribution perspective. Shanghai (0.017) ranked first in the number of public health institutions and three provinces (Tibet, Qinghai, and Inner Mongolian) had even less than 0.001 institutions per square kilometer. The technical personal number in Shanghai was nearly 1403 times than that in Tibet. Besides, Tibet also had the smallest number of beds among 31 provinces.

### The equality of public health resources allocation based on population

**Fig. 1:**
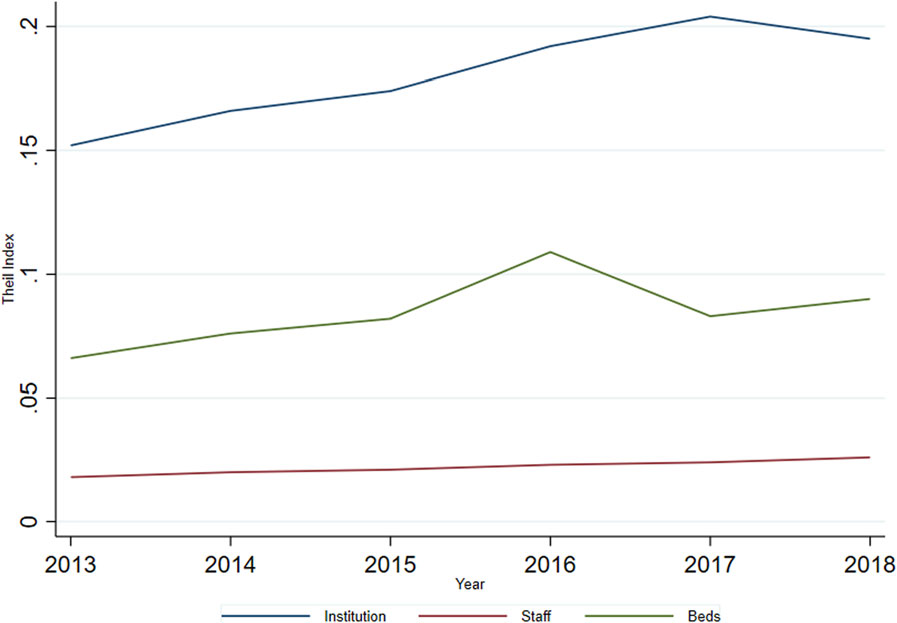
Theil index of public health resources allocation from 2013 to 2018

**Fig. 2:**
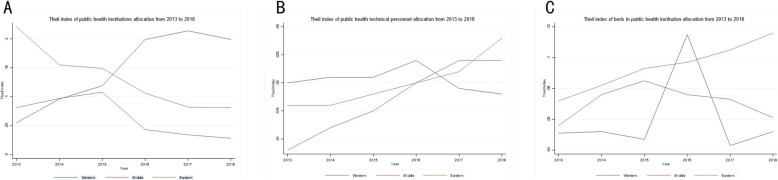
Theil index of three public health resources allocation from 2013 to 2018 (**a**, **b**, **c**)

The current situation of public health resource allocation demonstrateed that there were large gaps among 31 provinces in geographical-based allocation while the condition of population-based distribution was better. Overall, the public health resources concentrated in the eastern region and the middle region followed by. To future exploration, we adopted the Theil index to test the within- and between-equality in three regions, and the results are shown in Figs. 1 and 2.

Figure 1 is the trend of the Theil index changing from 2013 to 2018. The range of it was between 0.204 and 0.018. All three indicators had overall upward trends and the number of public health institutions was always higher than the other two resources though it droped from 0.204 to 0.195 in 2017. The Theil index of beds allocation also decreaseed in 2016 (from 0.115 to 0.083), but it rose again in 2017. Besides, the Theil index of technical personal was around 0.02.

The changes of the Theil index in three regions are reflected in Fig. 2. Theil index of eastern indicates that it continually growed in technical personnel (from 0.016 to 0.028) and beds allocation (from 0.072 to 0.116) from 2013 to 2018 and ranks first among three regions in 2018. While the western region showed a quite different situation – though its Theil index fluctuated during this period, it still experiences the highest equality in technical personnel (0.018) and beds (0.09) allocation in the same year. Comparing with other indicators, though the allocation of technical personal displayed an increasing trend, it still showed the highest fairness. The equality of public health institutions allocation sharply decreased, which was mainly contributed by the western region.

### Theil index contribution rate

**Fig. 3:**
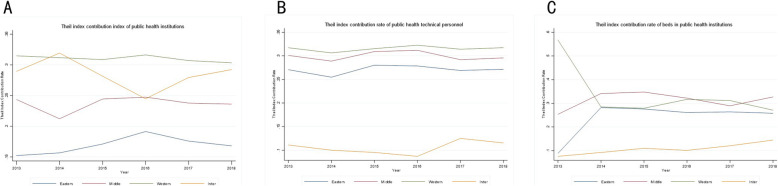
Contribution rate for Theil index of three public health resources allocation from 2013 to 2018 (**a**, **b**, **c**)

The Theil index contribution rates in the three regions is showed in Fig. 3. All regional contribution rates were larger than the inter-regional contribution rate except for public health institutions allocation. Among the three regions, the western region had the highest contribution rates and the beds allocation of that even beyond 50% in 2013. Also, the gap between inter- and intra-regional contribution in beds allocation was the smallest one in three indicators. The overall trend of the three regions’ contribution rates did not display significant difference from 2014 to 2018.

## Discussion

In this study, we found that the number of public health resources apart from public health institutions continually increased from 2013 to 2018, which in line with the growth of the population in China. Currently, the public health resources concentrated in the eastern region, and the population-based allocation gap was less than that in geographic-based allocation. Theil index results indicated that the population-based allocation equality of resources stably increased during this period. Inequality was mainly contributed by the western region.

The findings demonstrate that the regional distribution of public health technical personnel in 2018 is still under the basic requirement of the Chinese government plan. According to the Notice of the General Office of the State Council on Issuing the Outline for the Planning of the National Medical and Health Service System (2015–2020), the public health institutions and public health technical personal will reach 1 per 100,000 people and 0.83 per 1000 people in 2020, respectively [14]. More than half of the provinces in China achieve the goal of the public health institution plan. However, even in the province with the highest public health technical personnel per 10,000 people (Guangxi 7.33), the allocation of public health technical personnel is still less than plan.

Considering the measures in the Chinese health system reform, parts of the results are consistent with our expectancy. After 2013, the Chinese allocated funds in improving public health resources sustained. Because of policy support, the total public health resources increased. Besides, the continuing declines of public health institutions after 2015 was due to resource integration policy, where the National Health and Family Planning Commission of China (NHFPC) required local government to merge maternal and children health organization with family planning technical service institutions in district level [25]. The difference between population- and geographic-based allocation also have already been reported by other researches [15]. However, the results of equality evaluation show that though the Chinese government has a policy tendency towards western region such as increasing public health investment, staff training, etc., its contribution rate still increases. Also, the technical personnel and bed allocation equality in the eastern region are not optimistic.

Because of the Chinese national condition, the reason why policy tendency does not achieve the expected outcome can be explained. In China, technical personal are more willing to work in provincial capital or developed city, and this situation more serious in the western region [26]. The shortage of workforce may directly influence the survival of public health institutions in rural areas. Thus, although the government invests in the western regional public health system a lot, the Theil index contribution rates of public health institutions and technical personnel there still rise. Besides, investment inclination in the western region may imply the government ignores provinces in other regions. However, Table 2 has already shown that there is a huge gap in public health resources between Shanghai and Hebei, the number of whose resources even less than some western regional provinces. Thus, evidence-based allocation of public health resources-not only investment, but also human resources is important.

Some successful experiences in evidence-based resource allocation from other countries may have implications for this challenge. For instance, the Workload Indicators of Staffing Need, a demand-based approach used to distribute a package of health resources such as health staff, service, etc. based on the number of a given population, is proved effective in India, Uganda, and Zambia [27]. Furthermore, Australia’s government also builds a national platform to make sure to allocate the health workforce based on the community’s needs [28].

In summary, this study demonstrates the trend and current situation of public health resources allocation in China, as well as the main cause of fairness. However, there are still some limitations in this research: because of the available data, the public health investment is not included in the analysis, so the results can not reflect the whole picture of public health allocation. Besides, further researches need to be done to explore other factors that might influence the observation.

## Conclusion

In this study, we investigated the development and the current situations of Chinese public health resources. The results demonstrated that the amount of public health technical personal, beds, and equipment in public health institutions showed growth trends while the number of public health institutions decreased because of policy. From 2013 to 2018, the allocation inequality of public health resources stable increased, most of which was contributed by the western region.

All the evidence proved by this research is helpful for the government to provide investment to different regions. Further researches are needed to explore other influence factors of public health resource allocation.

## Data Availability

The datasets (China Statistical Yearbook 2019) generated and/or analyzed during the current study are available in the National Bureau of Statistics repository, http://www.stats.gov.cn/tjsj/ndsj/ The datasets (China Health Statistics Yearbook 2014 to 2019) generated and/or analyzed during the current study are available in the National Health Commission of the People’s Republic of China repository, http://www.nhc.gov.cn/zwgk/tjnj1/ejlist.shtml

## Abbreviations

SARS: severe acute respiratory syndrome
CHCs: community health centers
EBPHS: Basic Public Health Services
GDP: Gross Domestic Product
NHFPC: National Health and Family Planning Commission of China

## References

[1] Hoss A, Menon A, Corso L (2016). State public health enabling authorities: results of a fundamental activities assessment examining Core and essential services. J Public Health Manag Pract.

[2] Lee L (2004). The current state of public health in China. Annu Rev Public Health.

[3] Qingyue M, Hongwei Y, Wen C, Qiang S, Xiaoyun L (2015). People's Republic of China Health System Review. Vol.5 No.7.

[4] Yang L, Sun L, Wen L, Zhang H, Li C, Hanson K (2016). Financing strategies to improve essential public health equalization and its effects in China. Int J Equity Health.

[5] Hsiao WC (1995). The Chinese health care system: lessons for other nations. Soc Sci Med.

[6] Duckett J (2011). The Chinese state’s retreat from health: policy and the politics of retrenchment.

[7] Ma X, Wang H, Yang L, Shi L, Liu X (2019). Realigning the incentive system for China's primary healthcare providers. BMJ..

[8] Li C, Sun M, Wang Y, Luo L, Yu M, Zhang Y (2016). The Centers for Disease Control and Prevention system in China: trends from 2002–2012. Am J Public Health.

[9] Wang L, Wang Z, Ma Q, Fang G, Yang J (2019). The development and reform of public health in China from 1949 to 2019. Glob Health.

[10] Wang L, Liu J, Chin DP (2007). Progress in tuberculosis control and the evolving public-health system in China. Lancet..

[11] Yip W, Hsiao WC (2008). The Chinese health system at a crossroads. Health Aff (Millwood).

[12] Yuan B, Balabanova D, Gao J, Tang S, Guo Y (2019). Strengthening public health services to achieve universal health coverage in China. BMJ..

[13] Chinese State Council. General Office of the State Council on Printing and Distributing Deepening the Reform of the Medical and Health System Notice of Major Work Arrangements in 2013 [Internet]. Beijing: Chinese State Council;2013 [cited data: July 24^th^ 2020]. Available from: http://www.gov.cn/zwgk/2013-07/24/content_2454676.htm.Chinese..

[14] Chinese State Council. Notice of the General Office of the State Council on Issuing the Outline for the Planning of the National Medical and Health Service System (2015–2020). Beijing: Chinese State Council;2015 [cited data: July 24^th^ 2020]. Available from: http://www.gov.cn/zhengce/content/2015-03/30/content_9560.htm.Chinese.

[15] Liu W, Liu Y, Twum P, Li S (2016). National equity of health resource allocation in China: data from 2009 to 2013. Int J Equity Health.

[16] Zhang Y, Wang Q, Jiang T, Wang J (2018). Equity and efficiency of primary health care resource allocation in mainland China. Int J Equity Health.

[17] Cao Y, Shan J, Gong Z, Kuang J, Gao Y. Status and Challenges of Public Health Emergency Management in China Related to COVID-19. Front Public Health. 2020;8(250):250. 10.3389/fpubh.2020.00250 PMC7273973 32574311

[18] Fernandes Antunes A, Jacobs B, de Groot R, Thin K, Hanvoravongchai P, Flessa S (2018). Equality in financial access to healthcare in Cambodia from 2004 to 2014. Health Policy Plan.

[19] Tao Y, Henry K, Zou Q, Zhong X (2014). Methods for measuring horizontal equity in health resource allocation: a comparative study. Health Econ Rev.

[20] Regidor E (2004). Measures of health inequalities: part 1. J Epidemiol Community Health.

[21] National Bureau of Statistics of China. The categories of Chinese area China [Internet]. Beijing: National Bureau of Statistics of China; 2011 [cited data: July 24^th^ July]. Available from: http://www.stats.gov.cn/ztjc/zthd/sjtjr/dejtjkfr/tjkp/201106/t20110613_71947.htm.Chinese.

[22] Chinese State Council. Notice of the General Office of the State Council on Issuing the Outline for the Planning of the National Medical and Health Service System (2015–2020) [Internet]. Beijing:Chinese State Council;2015 [cited data: 2020 24^th^ July]. Available from: http://www.gov.cn/zhengce/content/2015-03/30/content_9560.htm.Chinese.

[23] Tyagarupananda S, Chattopadhyay N, Dasgupta I, Mitra M (2019). A generalization of the Theil measure of inequality. Deprivation, inequality and polarization: essays in honour of Satya Ranjan Chakravarty.

[24] Hosseinpoor AR, Bergen N, Barros AJ, Wong KL, Boerma T, Victora CG (2016). Monitoring subnational regional inequalities in health: measurement approaches and challenges. Int J Equity Health.

[25] Beijing Mental Health and Family Plan Commission. Implementation Opinions on Optimizing and Integrating Maternal and Child Health Care and Family Planning Technical Service Resources [Internet]. Beijing: Mental Health and Family Plan Commision. 2016 [cited data: 2020 24^th^ July]. Available from: http://www.beijing.gov.cn/zfxxgk/110088/lnfyc23/2016-09/05/content_735152.shtml.Chinese.

[26] Zhu B, Fu Y, Liu J, He R, Zhang N, Mao Y (2018). Detecting the priority areas for health workforce allocation with LISA functions: an empirical analysis for China. BMC Health Serv Res.

[27] Walsh FJ, Musonda M, Mwila J, Prust ML, Vosburg KB, Fink G (2017). Improving allocation and management of the health workforce in Zambia. Health Aff (Millwood)..

[28] Crettenden IF, McCarty MV, Fenech BJ, Heywood T, Taitz MC, Tudman S (2014). How evidence-based workforce planning in Australia is informing policy development in the retention and distribution of the health workforce. Hum Resour Health.

